# Essential Factors for Incompatible DNA End Joining at Chromosomal DNA Double Strand Breaks *In Vivo*

**DOI:** 10.1371/journal.pone.0028756

**Published:** 2011-12-14

**Authors:** Hideaki Ogiwara, Takashi Kohno

**Affiliations:** Division of Genome Biology, National Cancer Center Research Institute, Tokyo, Japan; National Institute on Aging, United States of America

## Abstract

Non-homologous end joining (NHEJ) is a major pathway for the repair of DNA double strand break (DSBs) with incompatible DNA ends, which are often generated by ionizing irradiation. *In vitro* reconstitution studies have indicated that NHEJ of incompatible DNA ends requires not only the core steps of synapsis and ligation, employing KU80/DNA-PKcs and LIG4, but also additional DNA end processing steps, such as DNA end resection by Artemis and gap-filling by POLλ and POLμ. It seems that DNA end processing steps are important for joining of incompatible DNA ends rather than compatible ends. Despite the fact that DNA end processing is important for incompatible DNA end joining *in vitro*, the role of DNA processing in NHEJ of incompatible DSBs *in vivo* has not yet been demonstrated. Here we investigated the *in vivo* roles of proteins implicated in each step of NHEJ using an assay in which NHEJ of incompatible DNA ends on chromosomal DNA can be assessed in living human cells. siRNA- or inhibitor-mediated impairment of factors in each NHEJ step resulted in a reduction in joining efficiency. Strikingly, stronger effects were observed when DNA end resection and ligation protein functions were impaired. Disruption of synapsis by KU80 and DNA-PKcs impairment, or the disruption of gap filling by POLλ and POLμ depletion, resulted in higher levels of microhomology-mediated joining. The present study indicates that DNA end resection and ligation factors are critical for the efficient joining of incompatible ends *in vivo*, further emphasizing the importance of synapsis and gap-filling factors in preventing illegitimate joining.

## Introduction

Non-homologous end joining (NHEJ) is a system that repairs DNA double strand breaks (DSB) by joining two broken DNA ends without requiring long stretches of homology, while homologous recombination repair (HRR) joins two broken DNA ends by using long (>100 bp) stretches of nucleotide homology [1], [2], [3]. In NHEJ, a complex consisting of the KU70/KU80 heterodimer and DNA-PKcs mediates the synapsis of two broken DNA ends and is followed by a ligation reaction performed by LIG4/XRCC4/XLF. NHEJ of compatible DNA ends requires synapsis and ligation factors in reconstitution systems, in which purified proteins and naked DNAs are reacted *in vitro*. Thus, these synapsis and ligation factors are defined as the core factors of NHEJ [1], [3], [4], [5]. The roles of synapsis and ligation factors in NHEJ have also been examined *in vivo* and it was found that the depletion of these factors reduces the efficiency of NHEJ, supporting the *in vitro* findings. Lack of synapsis factors prompts micro-homology–mediated joining, an alternative mode of NHEJ that does not require synapsis factors [6], [7], [8], [9].

Other factors involved in NHEJ include DNA end resection and/or gap-filling proteins that process the DNA ends to be joined. Pathological and physiological DSBs, such as those generated by ionizing radiation (IR), often leave incompatible DNA ends that require such processing before joining [1], [4], [10]. Therefore, DNA end resection and gap-filling are likely to be additional but critical steps for NHEJ of DSBs *in vivo*. So far, the molecular processes of DNA end resection and gap-filling have been exclusively investigated using reconstitution systems *in vitro*. Artemis, a DNA nuclease, [11], and two DNA polymerases (POLλ and POLμ) [12], [13] were implicated in end resection and gap filling, respectively. However, whether these proteins contribute to NHEJ repair of DSBs *in vivo* remains unknown due to the lack of appropriate cell-based assay systems in which the function of DNA end processing factors in NHEJ can be monitored.

In the present study, we investigated the *in vivo* roles of NHEJ factors involved in synapsis, DNA end resection, gap-filling or ligation by using an assay that we recently developed in which NHEJ against chromosomal DSBs with incompatible DNA ends can be assessed in living human cells [14]. In this system, DSBs were generated by I-*Sce*I endonuclease, which results in incompatible DNA ends that require DNA end resection and gap filling in order to be joined. The efficiency of repair was evaluated by FACS analysis of eGFP protein produced from the joined products. Modes for joining were deduced by sequencing of the breakpoint junctions of the joined products. Therefore, we were able to determine the *in vivo* contribution of each protein to NHEJ by depleting or inhibiting each factor and subsequently examining its effect on the efficiency and mode of joining.

## Results

### NHEJ efficiency of chromosomal DSBs with incompatible DNA ends

**Figure 1:**
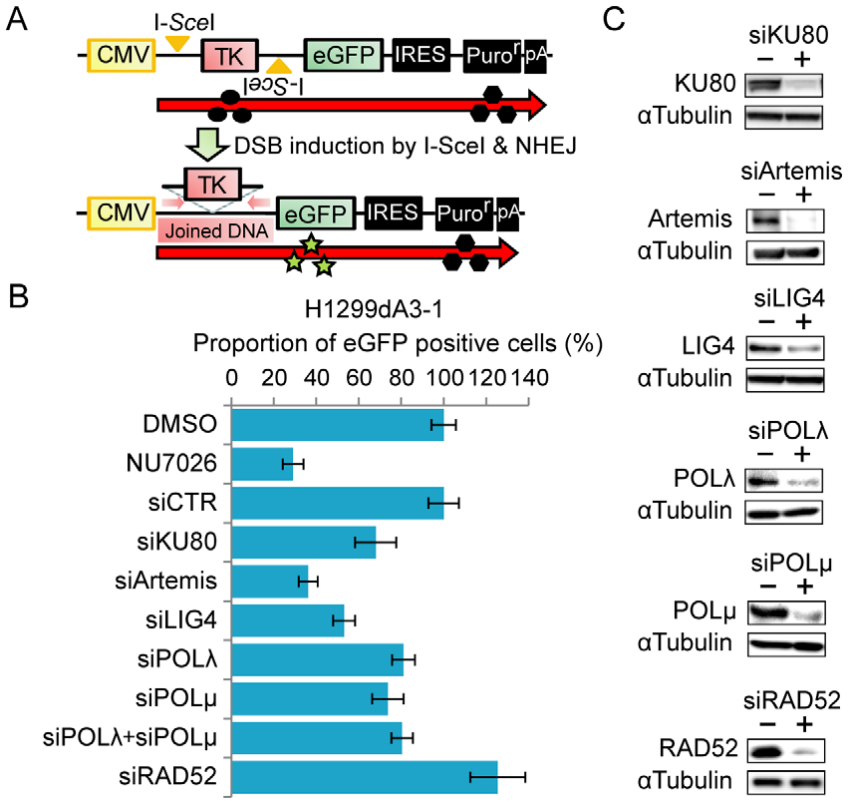
End-joining efficiency is reduced when NHEJ factors are impaired. (**A**) Scheme of the assay. Two I-*Sce*I sites in reverse orientation are indicated by yellow arrow heads. The locations of the PCR primers used for the amplification of joined products are indicated by the red arrows. CMV: cytomegalovirus promoter/enhancer; IRES: internal ribosome entry site; pA: polyA signal. (**B, C**) siRNA- or inhibitor-mediated impairment of NHEJ proteins reduces NHEJ efficiency. (B) The results obtained 48 hr after the transfection of the I-*Sce*I expression plasmid are shown. The proportion of eGFP-positive cells treated with siRNA or NU7026 (50 uM) is expressed as a ratio of values from siRNA-treated cells versus cells treated with non-targeting siRNA (siCTR) or DMSO. (C) The results of immunoblot analysis.

**Figure 2:**
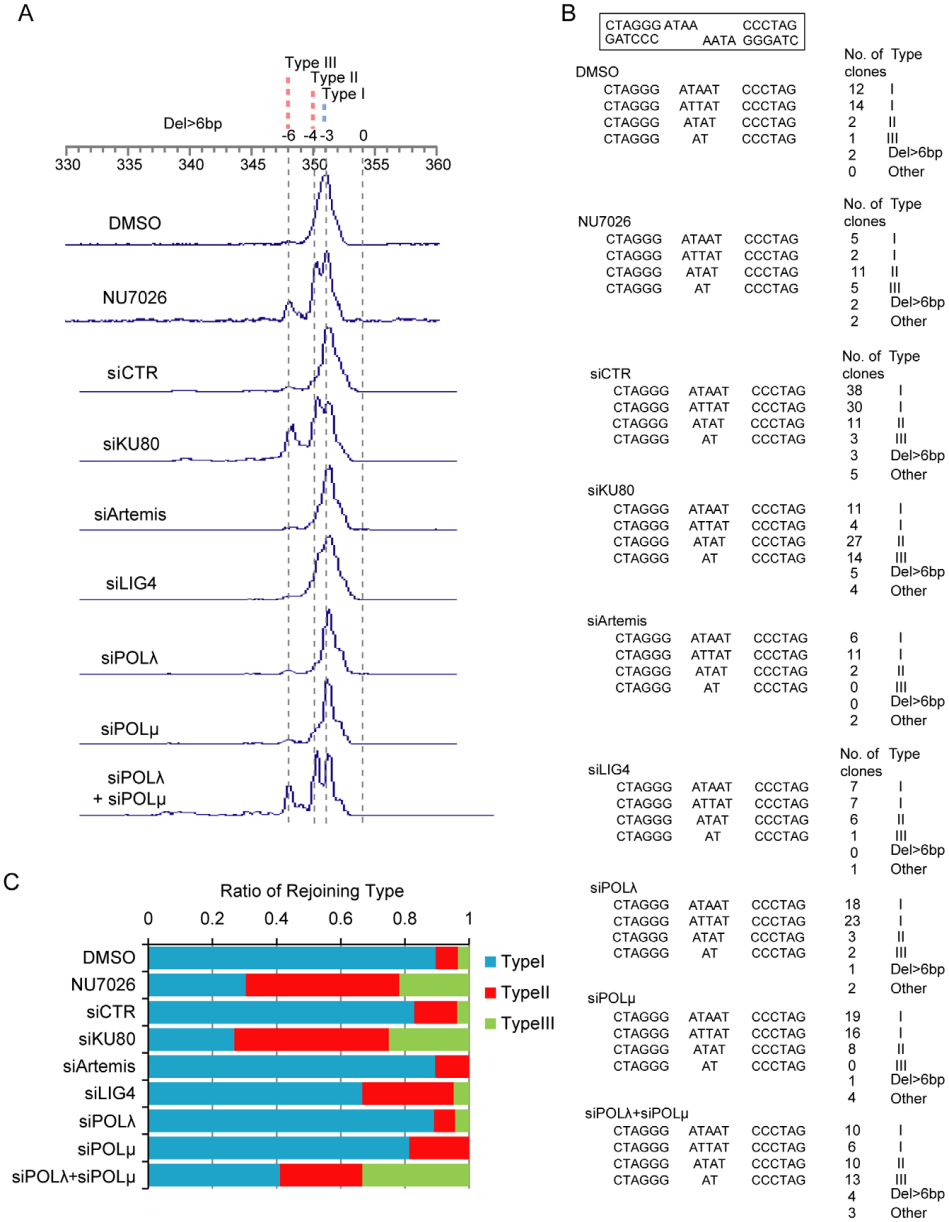
Structure of breakpoint junctions from NHEJ impaired cells. (**A**) Size fractionation of DNA fragments containing breakpoint junctions. The sizes of Type I–III products are shown on top. (**B, C**) Nucleotide sequences of the breakpoint junctions from NHEJ-impaired cells. (**B**) The sequences of the breakpoint junctions with the clone number and product type. The structure of the DNA ends generated by I-*Sce*I is shown in the rectangle. (**C**) Type I, II and III products according to NHEJ protein impairment (shown as ratios after removing “Del>6bp” and “Other” products).

To monitor NHEJ efficiency of chromosomal DSBs with incompatible DNA ends *in vivo*, we employed a recently described cell-based assay system (**Figure 1A**) [14]. Briefly, a repair substrate containing two I-*Sce*I sites was integrated into the chromosomal DNA of H1299 human lung cancer cells. NHEJ of two broken DNA ends generated by I-*Sce*I endonuclease digestion results in the deletion of the HSV-TK (HSV-thymidine kinase) open reading frame and leads to the production of a transcript that enables the translation of enhanced green fluorescent protein (eGFP) instead of the HSV-TK protein. Therefore, the level of NHEJ activity in living cells can be evaluated by the proportion of eGFP-positive cells. The two DNA ends produced on chromosomal DNA are incompatible (see **Figure 2B**) since the two I-*Sce*I sites were integrated in opposite directions (**Figure 1A**) and are predicted to be joined by NHEJ, which may include the end resection and gap filling steps.

We examined the NHEJ efficiency of cells with defects in each of the NHEJ steps by disrupting the function of the following factors: KU80 and DNA-PKcs (synapsis), Artemis (DNA end resection), POLλ and POLμ (gap filling), and LIG4 (ligation). KU80, Artemis, LIG4, POLλ and POLμ were depleted by RNAi with similar efficiency (**Table S1**). DNA-PKcs was impaired by a 50 µM concentration of NU7026, a specific DNA-PKcs inhibitor previously shown to induce radiosensitization of cancer cells [14], [15], [16]. siRNA-mediated depletion or drug-mediated inhibition of the NHEJ factors significantly decreased the GFP-positive cell fractions (**Figure 1B, 1C** **, Figure S1**). In contrast, depletion of RAD52, which is involved in homology-mediated repair [2], did not cause such a reduction. Thus, not only the proteins involved in the core steps of NHEJ (synapsis and ligation) but also those involved in DNA end processing (DNA end resection and gap filling) affect end joining efficiency. Particularly, the depletion of Artemis and LIG4 led to a drastic reduction in end joining, demonstrating their important contribution to NHEJ *in vivo*.

### NHEJ modes of incompatible DNA ends

**Figure 3:**
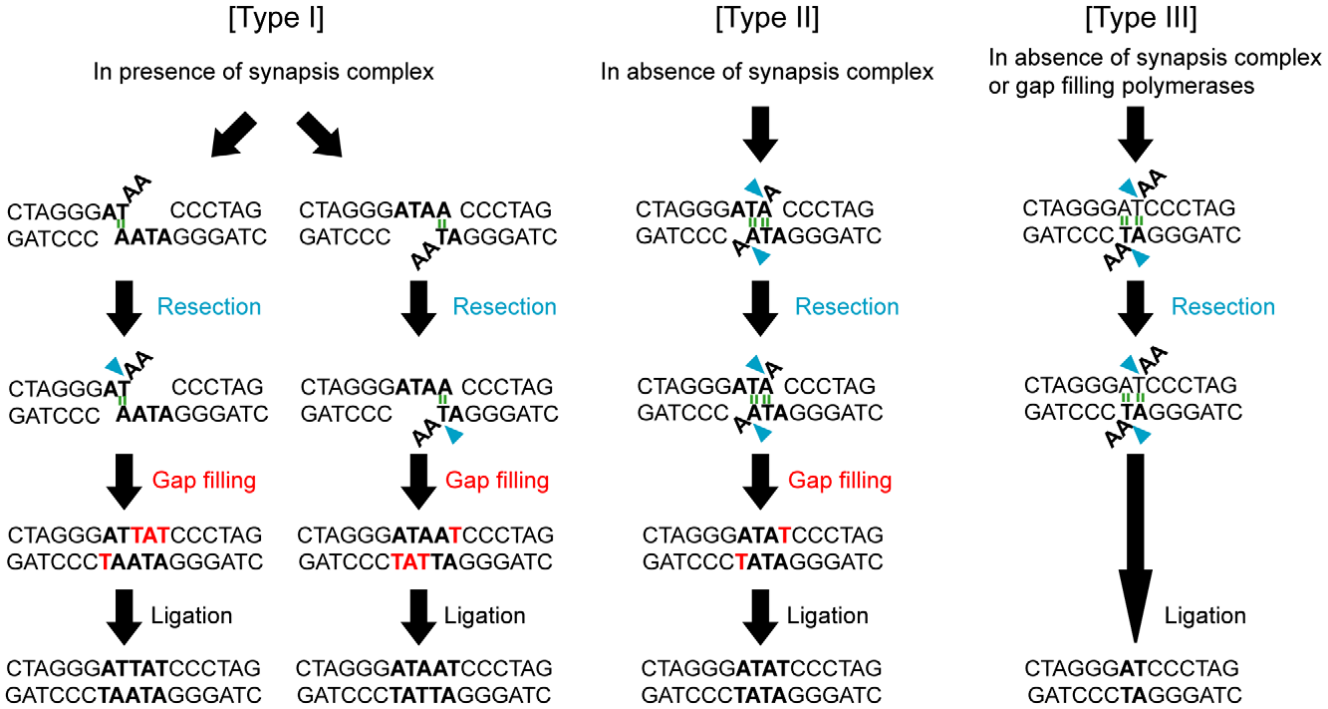
Deduced NHEJ process for the formation of joint products. (**A**) Type I products are formed by joining, which is accompanied with the resection of two bases at one DNA end, followed by gap filling and ligation. (**B**) Type II products are formed by joining, which is accompanied by the resection of one base at both DNA ends, followed by gap filling and ligation. (**C**) Type III products are formed by joining, which is accompanied by the resection of two bases at both DNA ends, followed by ligation. The type I product is formed predominantly in the presence of synapsis proteins. Type II and III products are predominantly formed in the absence of synapsis proteins. Hydrogen bonds are likely used to anneal the DNA ends (green). Type III products are formed via the annealing of DNA ends using a two base homology pair without gap filling, and are thus a major product formed in the absence of gap-filling proteins POLλ and POLμ.

To deduce the mode of end joining from the nucleotide sequences of the breakpoint junctions, joined products from cells with/without impairment of NHEJ proteins were cloned, PCR amplified, size fractionated and sequenced. Three major recurrent types of products (Types I–III) were observed, as well as several other types. The 351 bp Type I product was the predominant form detected in cells transfected with non-targeting siRNA (siCTR) or DMSO (solvent treatment) (**Figure 2A–C**). This product was deduced to be formed by the joining of the DNA ends, which is accompanied with a two base resection at one DNA end and followed by gap filling and ligation (**Figure 3A**).

Fractions of formed products were significantly (*P*<0.05 by exact test) affected by the impairment of KU80 or DNA-PKcs (NU7026 treatment) but not by that of Artemis, LIG4, POLλ or POLμ (**Table S2**). Notably, smaller sized products, specifically Type II (350 bp) and Type III (348 bp), were predominant in KU80-depleted or NU7026-treated cells compared with control cells (**Figure 2A–C**). Type II and Type III products exhibited the loss of 1 and 3 more base pairs than Type I products. This result is consistent with previous results showing that KU70/KU80 and DNA-PKcs play a major role in synapsis, since the Type II and III products were likely formed by joining using 2 bp microhomology, instead of synapsis, followed by the resection of one and two bases at both DNA ends, respectively (**Figure 3B–C**).

In Artemis- or LIG4-depleted cells, the spectrum of formed products did not differ significantly from that of cells transfected with non-targeting siRNA (**Figure 2A–C**), which is consistent with the fact that end resection and ligation were common among all three types of products (**Figure 3**). The results were also consistent with our previous result showing that Artemis or LIG4 depletion resulted in a stronger reduction of joining efficiency than the depletion of other NHEJ factors (**Figure 1B**).

In either POLλ- or POLμ-depleted cells, fractions of formed products also did not differ significantly from those in cells transfected with non-targeting siRNA. However, POLλ and POLμ double depletion caused a significant difference in the fraction of products (*P* = 5.2×10^−6^ by exact test), with Type III (348 bp) products being the predominant form detected. This type of product was deduced to be formed without gap filling (**Figure 3C**). Therefore, this result strongly indicates that POLλ and POLμ play redundant roles in gap filling in NHEJ.

## Discussion

Here, we showed that the impairment of not only synapsis and ligation factors but also DNA end resection and gap filling factors decreased the efficiency of end joining in our cell-based assay system. Previously, the molecular mechanism of incompatible DNA end joining, including the DNA end processing steps, had been studied only using reconstitution experiments *in vitro*. Therefore, we have shown for the first time *in vivo* that all steps of NHEJ (synapsis, ligation, end resection and gap filling) contribute to efficient incompatible DNA end joining. The depletion of Artemis or LIG4 led to a greater reduction in joining efficiency than that of other factors. DNA end resection is an inevitable event for the joining of incompatible DNA ends. Therefore, DNA end resection factors may be necessary for efficient end joining in addition to ligation proteins. Impairment of DNA-PKcs by NU7026 treatment resulted in a strong inhibition of end joining, while siRNA-mediated DNA-PKcs depletion also led to a reduction in joining efficiency to the same degree as Artemis depletion did (data not shown). Interestingly, DNA-PKcs was reported to be required for the nuclease activity of Artemis [17]. Therefore, the strong inhibitory effect of DNA-PKcs impairment on joining efficiency might be due to its role in inhibiting DNA end resection.

We showed that the impairment of synapsis factors KU80 and DNA-PKcs led to end joining with more DNA end resection in human cells, consistent with the previous study involving rodent cells [18]. siRNA-mediated depletion of KU80 was inferred to impair the KU70/KU80 complex also by de-stabilizing KU70 protein [19]. KU70/KU80 forms a synapsis complex with DNA-PKcs, which brings both DNA ends together, and the incompatible DNAs with flap ends are subsequently processed by end resection and gap filling proteins to generate compatible DNA ends [20], [21]. An *in vitro* reconstitution study indicated that, in the absence of synapsis factors, DNA ends can be joined by four or more hydrogen bonds (two or more base pairs) [12]. Types II and III products were likely formed through the annealing of DNA ends by four hydrogen bonds using 2 bp micro-homology, and the impairment of KU80 and DNA-PKcs led to a predominance of Type II and Type III product formation. Thus, our results indicate that a synapsis-independent microhomology-mediated joining occurs *in vivo*, as previously suggested by the *in vitro* data.

Our study indicates that the impairment of gap filling factors POLλ and POLμ also affect the mode of joining. Depletion of POLλ and POLμ together, but not individually, primarily resulted in Type III product formation; thus, the incompatible DNA ends were likely processed to generate compatible ends without gaps. Previous *in vitro* studies have indicated the significance of POLλ and POLμ in incompatible end joining [22], [23]. Particularly, one study using a reconstitution system *in vitro* clearly showed that POLλ and POLμ contributed co-operatively, but not competitively, to gap filling in the joining of incompatible DNA ends [12]. Our result indicates that POLλ and POLμ contribute redundantly to the gap filling process *in vivo*, which is consistent with the above mentioned studies *in vitro*. We demonstrated here that the mode of joining incompatible DNA ends is determined not only by synapsis factors but also by gap filling factors. To the best of our knowledge, this is the first report demonstrating the redundant contribution of POLλ and POLμ to NHEJ *in vivo*. Interestingly, POLμ has been suggested to contribute not only to gap-filling but also to end-bridging [23]. Therefore, reduction in joining efficiency following POLλ and POLμ depletion might be also due to the loss of end-bridging activity, which is an issue that will be investigated in future studies.

The *in vivo* assay system used here was useful for the analysis of the molecular mechanisms of incompatible DNA end joining. In the present study, only representative proteins were analyzed. However, other DNA polymerases, such as POL β, are also suggested to be involved in gap filling in *in vitro* experiments [12], [24]. In addition, chromatin remodelers, such as covalent remodelers (histone acetyltransferases, CBP/p300 and TIP60; and histone deacetylases, HDAC1 and HDAC2) and non-covalent ATPase dependent remodelers (SWI/SNF complex and ACF complex proteins) have been also been reported to participate in this process [14], [25], [26], [27], [28]. A comprehensive depletion analyses of each of those genes, individually and in combination, will elucidate the molecular mechanisms of joining of incompatible DNA ends. A potential limitation of the present study is that only a single type of incompatible end was examined for joining. In fact, depending on the composition of incompatible DNA ends, POLλ and POLμ have been suggested to differentially contribute to the joining of incompatible ends [24], [29], although no difference between POLλ and POLμ in this respect was observed in this study. Therefore, analyses of the joining of several types of DNA ends will further provide us with valid and detailed information about the individual contributions of POLλ and POLμ to end-joining.

Finally, our study defined both the core and non-core DNA end processing NHEJ factors that are responsible for NHEJ activity and mode selection. Particularly, abrogation of not only synapsis but also gap filling factors primarily resulted in microhomology-mediated end joining. This type of joining is representative of an illegitimate repair pathway resulting in the loss of nucleotides at DSB ends and is thought to underlie genome instability in cancer cells, since breakpoint junctions of chromosomal interstitial deletions and translocations in cancer cells frequently retain traces of microhomology-mediated joining [30], [31], [32]. Large amounts of DSBs have been shown to occur in pre-malignant cells for human lung and other cancers [33], [34]. Therefore, such cells perform DSB repair as a way to survive from high levels of DNA damage. The accumulation of genetic alterations during carcinogenesis may be a result of the limited amount of synapsis and gap filling factors. In addition, NHEJ activity was significantly inhibited by the depletion of ligation and synapsis factors. Inhibitors of DNA-PKcs kinase activity sensitize cancer cells to IR and are awaiting evaluation for clinical application as a sensitizer in radiotherapy [35]. Inhibitors of proteins involved in the ligation and end resection steps might also be useful as radiosensitizers in cancer therapy.

## Methods

### NHEJ assay

H1299dA3-1#1, a clone of H1299 human lung cancer cells (obtained from Dr. John D. Minna of UT Southwestern Medical Center), stably carries the IRES-TK-EGFP DNA within their genome [14]. NHEJ assay was performed as described in [14]. Briefly, pCBASce plasmid (I-*Sce*I expression plasmid) DNA (0.8 µg) was introduced into 7.5×10^4^ of H1299dA3-1#1 cells per well in a 24-well plate by transfection with Lipofectamine 2000 reagent (Invitrogen, Carlsbad, CA). For FACS analysis, cells were harvested by trypsinization, washed with PBS, and applied to a FACS Calibur cytometer (Beckton Dickinson, Franklin Lakes, NJ). Fractions of eGFP positive cells were determined by three independent analyses and were expressed as means +/− standard deviations. To examine the effect of siRNAs on DNA joining, cells were subjected to the NHEJ assay and western blot analysis 48 hours after siRNA transfection. siRNAs were transfected at a concentration of 50 nM using Lipofectamine RNA MAX (Invitrogen). To examine the effect of NU7026 (50 µM) (Sigma, St Louis, MO) on NHEJ, either NU7026 or DMSO was added at the time of I-*Sce*I introduction until the samples were collected for analysis. A representative result of at least two independent experiments is shown for each depletion/inhibition.

### siRNA-mediated gene knock down

The following siRNA duplexes were purchased from Dharmacon (Lafayette, CO) or Qiagen (Valencia, CA): siCTR (D-001810-01), KU80 (J-010491-05), Artemis (SI00133945), POLλ (J-008746-05), POLμ (J-010035-09), LIG4 (J-004254-09) and RAD52 (J-011760-05). siRNAs were transfected into 2×10^4^ cells in a 12-well plate at a final concentration of 50 nM using lipofectamine RNA MAX (Invitrogen). At 72 hours after siRNA transfection, the cells were subjected to the NHEJ assay as well as western blot analysis to examine knockdown efficiency. Protein levels in siRNA-treated cells were examined by western blot analysis using specific antibodies. Antibodies used in this study were purchased from Sigma (α-tubulin: T6199), Bethyl Laboratories (Artemis: A300-234A), Santa Cruz Biotechnology (POLλ: sc21531; POLμ: sc27769; KU80: sc9034; LIG4: sc11750), and Cell Signaling Technology (RAD52: #3425).

### Analysis of breakpoint junctions

DNA fragments containing breakpoint junctions were amplified by PCR using 10 ng of genomic DNAs obtained from H1299dA3-1#1 cells subjected to NHEJ assay as templates. To examine the size of the fragments, PCR was performed with a set of primers, BP-F* (FITC-labeled) and maxGFP-R2, and the PCR products were directly separated by electrophoresis using an ABI 3700 Sequence Analyzer (Applied Biosystems, Foster City, CA) and analyzed by the Gene Scan software. To determine the sequences of the breakpoint junctions, PCR products amplified by BP-F1 and maxGFP-R2 were subcloned into the pGEM-T vector (Promega, Madison, WI) by TA-cloning and were sequenced using the ABI 3700 Sequence Analyzer and the sequencer 4.7 software (Applied Biosystems). Nucleotide sequences of the BP-F1 and maxGFP-R2 primers were previously described [14]. The sequences of breakpoint junctions were determined for each depletion/inhibition by direct sequencing of colony-PCR products.

## Supporting Information

Figure S1 eGFP-positive cells (boxed) assessed by FACS analysis 48 hours after an I-SceI expression plasmid transfection.
(PDF)

Table S1 Knockdown efficiency.
(PDF)

Table S2 Joined products resulting from the impairment of NHEJ proteins.
(PDF)
