# Supplementary material for: Essential Factors for Incompatible DNA End Joining at Chromosomal DNA Double Strand Breaks In Vivo
Source: PLoS One. 2011 Dec 14;6(12):e28756. doi: 10.1371/journal.pone.0028756 (PMC3237495; doi:10.1371/journal.pone.0028756)
Supplement: Figure S1 — eGFP-positive cells (boxed) assessed by FACS analysis 48 hours after an I-SceI expression plasmid transfection. (PDF) [file pone.0028756.s001.pdf]

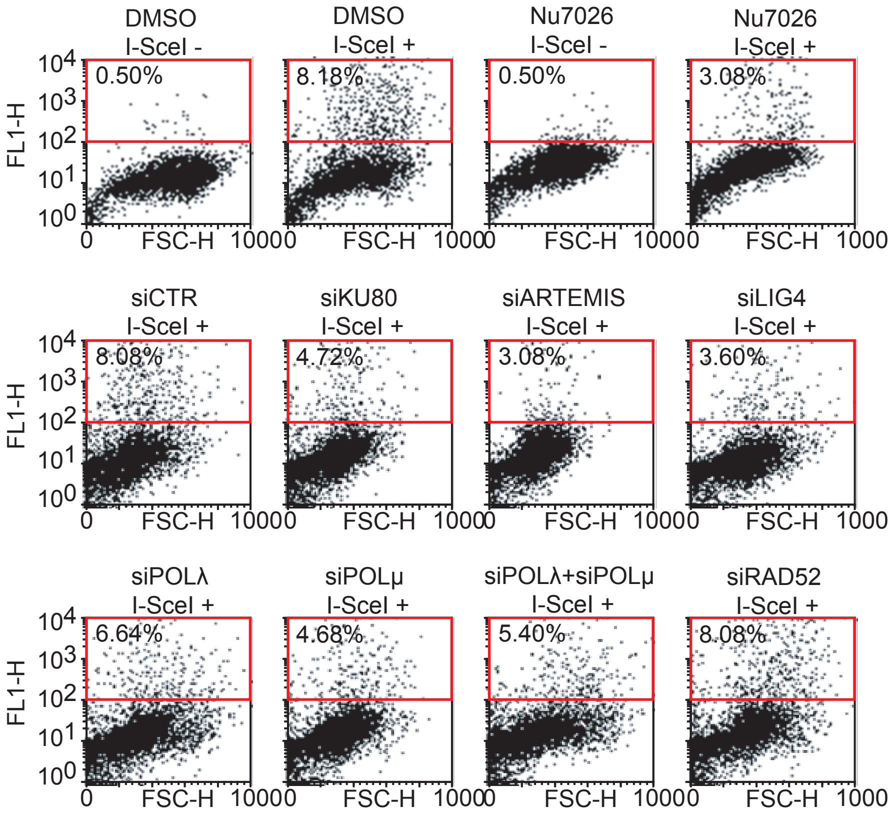

Figure S1.
eGFP-positive cells (boxed) assessed by FACS analysis 48 hours after an I-SceI expression plasmid transfection.
