# Supplementary material for: Essential Factors for Incompatible DNA End Joining at Chromosomal DNA Double Strand Breaks In Vivo
Source: PLoS One. 2011 Dec 14;6(12):e28756. doi: 10.1371/journal.pone.0028756 (PMC3237495; doi:10.1371/journal.pone.0028756)
Supplement: Table S1 — Knockdown efficiency. (PDF) [file pone.0028756.s002.pdf]

Table S1. Knockdown efficiency.

Target of RNA interference	Target protein fraction*
CTR	1.00
KU80	0.08
Artemis	0.15
LIG4	0.26
POLλ	0.24
POLμ	0.13
POLλ&POLμ	0.27/0.28
RAD52	0.16

* Fraction of protein levels from targeting siRNA-transfected cells versus those from non-targeting siRNA-transfected cells.
