# Supplementary material for: Essential Factors for Incompatible DNA End Joining at Chromosomal DNA Double Strand Breaks In Vivo
Source: PLoS One. 2011 Dec 14;6(12):e28756. doi: 10.1371/journal.pone.0028756 (PMC3237495; doi:10.1371/journal.pone.0028756)
Supplement: Table S2 — Joined products resulting from the impairment of NHEJ proteins. (PDF) [file pone.0028756.s003.pdf]

Table S2. Joined products resulting from the impairment of NHEJ proteins

	Product					P value by exact test against CTR/DMSO
	Type I	Type II	Type III	Del	Other	
DMSO	26	2	1	1	0	-
NU7026	7	11	5	2	2	1.9E-05
CTR	68	11	3	3	5	-
KU80	15	27	14	5	4	2.4E-10
Artemis	17	2	0	0	2	0.74
LIG4	14	6	1	0	1	0.41
POLλ	41	3	2	1	2	0.82
POLμ	35	8	0	1	4	0.71
POLλ&POLμ	16	10	13	4	3	5.2E-06
